# Psychometric Properties of the Hospital Anxiety and Depression Scale (HADS) in Previously Hospitalized COVID-19 Patients

**DOI:** 10.3390/ijerph19159273

**Published:** 2022-07-29

**Authors:** César Fernández-de-las-Peñas, Jorge Rodríguez-Jiménez, María Palacios-Ceña, Ana I de-la-Llave-Rincón, Stella Fuensalida-Novo, Lidiane L. Florencio, Silvia Ambite-Quesada, Ricardo Ortega-Santiago, José L. Arias-Buría, Bernard X. W. Liew, Valentín Hernández-Barrera, Margarita Cigarán-Méndez

**Affiliations:** 1 Department of Physical Therapy, Occupational Therapy, Rehabilitation and Physical Medicine, Universidad Rey Juan Carlos, 28922 Alcorcón, Spain; jorge.rodriguez@urjc.es (J.R.-J.); maria.palacios@urjc.es (M.P.-C.); anaisabel.delallave@urjc.es (A.I.d.-l.-L.-R.); stella.fuensalida@urjc.es (S.F.-N.); lidiane.florencio@urjc.es (L.L.F.); silvia.ambite.quesada@urjc.es (S.A.-Q.); ricardo.ortega@urjc.es (R.O.-S.); joseluis.arias@urjc.es (J.L.A.-B.); 2 School of Sport, Rehabilitation and Exercise Sciences, University of Essex, Colchester CO4 3SQ, UK; liew_xwb@hotmail.com; 3 Department of Public Health, Universidad Rey Juan Carlos, 28922 Alcorcón, Spain; valentin.hernandez@urjc.es; 4 Department of Psychology, Universidad Rey Juan Carlos, 28922 Alcorcón, Spain; margarita.cigaran@urjc.es

**Keywords:** Hospital Anxiety and Depression Scale, COVID-19, long COVID, validity

## Abstract

Severe acute respiratory syndrome coronavirus 2 (SARS-CoV-2) virus is associated with psychological/emotional disturbances. This study aimed to assess internal consistency, reliability, and construct validity of the Hospital Anxiety and Depressive Scale (HADS), as a patient-reported outcome measure (PROM) for evaluating emotional consequences of SARS-CoV-2 in hospitalized COVID-19 survivors with long COVID. The LONG-COVID-EXP-CM is a multicenter cohort study including patients hospitalized by COVID-19 during the first wave of the pandemic in five hospitals in Madrid. A total of 1969 (age: 61 ± 16 years, 46.5% women) COVID-19 survivors experiencing post-COVID symptoms a mean of 8.4 ± 1.5 months after hospital discharge completed HADS. Internal consistency (Cronbach α), reliability (item-internal consistency, item-discriminant validity), construct validity (confirmatory factor analysis), and floor effect and ceiling effect were calculated. The mean time for fulfilling HADS was 65 ± 12 s. A ceiling effect ranging from 1.99% to 13.74% and a floor effect ranging from 43.05% to 77.77% was observed. Based on the item-scale correlation coefficients, the Cronbach’s alpha values reflecting the internal consistency reliability were 0.890 for the anxiety scale (HADS-A) and 0.856 for the depressive scale (HADS-D) The correlation coefficient between HADS-A and HADS-D scores was excellent (r: 0.878). The confirmatory factor analysis revealed that five out of the seven fitness indexes were excellent: CFI = 0.969, NNFI = 0.963; TLI = 0.963; AGFI = 0.951; GFI = 0.972), supporting good construct validity. In conclusion, this study indicates that both anxiety and depressive symptoms scales of HADS had overall good psychometric properties to be used for assessing psychological and emotional stress in COVID-19 survivors with long COVID.

## 1. Introduction

Severe acute respiratory syndrome coronavirus 2 (SARS-CoV-2) virus, responsible for causing coronavirus disease 2019 (COVID-19), mainly affects the respiratory system; however, multisystem effects associated with the development of a plethora of symptoms is also present in most patients [1]. As well as biologically related symptoms, emotional symptoms have been also reported by these patients which supports the observation that biological and behavioral factors interact in COVID-19 context [2]. For instance, the prevalence of anxiety and depressive symptoms has been reported to be 38% in people affected by SARS-CoV-2 virus [3]. Furthermore, anxiety and depressive symptoms are also prevalent after the acute phase of the infection (post-COVID phase) [4]. Interestingly, Bottemanne et al. recently reported that depressive levels after an acute COVID-19 episode are associated with an increased risk of physical post-COVID symptoms such as pain and dyspnea [5].

In addition, the prevalence of anxiety and depressive symptoms has also increased in people not infected by COVID-19. For instance, the prevalence of anxiety/depressive levels in the general population after the COVID-19 outbreak was higher when compared with pre-pandemic data [6]. Similarly, up to 18% of relatives [7] or 38% of healthcare professionals (i.e., nurses) [8] attending to COVID-19 patients also exhibited high levels of anxiety and depression. Therefore, evaluation of anxiety or depressive levels within the COVID era is essential.

Patient-reported outcome measures (PROM) consist of generic or disease-specific self-reported questionnaires evaluating different aspects of a particular condition. Some disease-specific PROMs such as the post-COVID-19 functional status scale (PCFS) have been developed for evaluating the functional capabilities of COVID-19 survivors [9,10]. However, this PROM omits the emotional and psychological aspect of the condition [9,10].

The Hospital Anxiety and Depression Scale (HADS) was originally developed in 1983 to identify the presence of anxiety disorders and depressive symptoms in people in nonpsychiatric hospital clinics [11]. Bjelland et al. found good psychometric properties of both the anxiety and depression scales of HADS for assessing for anxiety disorders and depressive symptoms within the general population and also in psychiatric patients [12]. More recently, studies have confirmed the internal consistency and validity of HADS in a general population aged 65–80 years old [13].

HADS is the PROM most used in COVID-19 research for evaluating anxiety and depressive symptoms [3,4]. However, the psychometric properties in the COVID-19 context has not been properly investigated. Tasnim et al. found that HADS had good psychometric properties for evaluating anxiety and depressive levels in healthcare workers who were in the frontline during the first COVID-19 outbreak [14]. No previous study has evaluated the psychometric properties of HADS in people with long COVID. The current study aimed to describe internal consistency, reliability, and construct validity of HADS in previously hospitalized COVID-19 survivors.

## 2. Methods

### 2.1. Participants

An instrumental research design evaluating the psychometric data of HADS were used [15]. We used data collected from a multicenter cohort study (LONG-COVID-EXP-CM) including patients hospitalized by COVID-19 during the first wave of the pandemic (from 10 March to 31 May 2020) in five urban hospitals of Madrid (Spain). All diagnoses were conducted with real-time reverse transcription-polymerase chain reaction (RT-PCR) assay of nasopharyngeal/oral swab samples as well as clinical and radiological findings at hospital admission. Patients discharged from the involved hospitals were included in an anonymous database and a random selection of 400 patients from each hospital was performed with randomization software. This multicenter study design was approved by the Local Ethics Committees of all the involved institutions (URJC0907202015920, HCSC20/495E, HUFA 20/126, HUIL/092-20, HUF/EC1517, HSO25112020). Participants provided their informed consent before collecting any data. It should be noted that the participants from the LONG-COVID-EXP-CM study have been referred to in previous letters to the editor or publications [16,17,18,19,20,21] but current data presented here are completely new and not previously published.

### 2.2. COVID-19 and Post-COVID Data Collection

Demographic (e.g., age, gender, height, weight), clinical (e.g., previous medical comorbidities), and hospitalization (COVID-19 associated-onset symptoms experienced at hospital admission, intensive care unit admission, days at hospital) data were collected from hospital records. Participants were scheduled for a telephone interview conducted by healthcare researchers and were systematically asked about the presence of symptoms that they experienced after hospital discharge (post-COVID-19 related symptoms). A predefined list of symptoms including dyspnea, fatigue, throat pain, cough, anosmia, ageusia, hair loss, skin rashes, diarrhea, palpitations, brain fog, concentration loss, or ocular disorders was used, but participants were free to report any symptom not included in the list and experienced at the time of the assessment.

### 2.3. Hospital Anxiety and Depression Scale (HADS)

Both the anxiety (HADS-A) and depression (HADS-D) subscales of HADS were assessed. It has been found that HADS can be properly evaluated by telephone [22]. HADS-A consists of 7 items assessing anxiety symptoms whereas HADS-D consists of 7 items evaluating depressive symptoms. Each item is scored on a 4-point Likert scale (0–3) providing a maximum of 21 points for each subscale [23]. We applied a cut-off score of ≥8 points for each scale since this value has shown good sensitivity and specificity to determine the presence of anxiety or depressive symptoms, respectively [24].

### 2.4. Statistical Analysis

Statistical analysis was performed with STATA 16.1 program (StataCorp. 2019. Stata Statistical Software: Release 16. College Station, TX: StataCorp LP. USA). Proportions, means, and standard deviations were used to describe the study population. Since all items from HADS are answered into a Likert scale, we present the percentage of those individuals answering each item. Statistical significance was defined when *p*-value < 0.05. We tested internal consistency, reliability, and construct-validity properties of HADS according to the consensus-based standards for the selection of health measurement instruments (COSMIN) [25].

The Cronbach’s alpha [26] and the Raykow Omega coefficient [27] were calculated to determine internal consistency, where values between 0.7 and 0.95 were considered as good internal consistency. Reliability of HADS was evaluated by calculating item-internal consistency, i.e., testing the correlation between each item and its hypothesized scale with correction for overlap. A correlation coefficient of 0.4 supported item-internal consistency. Furthermore, item-discriminant validity was supported if the correlation between an item and its hypothesized scale was higher than its correlation with all other scales. Construct validity was examined with a confirmatory factor analysis (CFA). The following packages were used: lavaan [28] for CFA analysis and semPlot [29] for visualizing the CFA paths. CFA was first used to assess the fit of the proposed measurement model using a two-factor model (anxiety, and depression). The weighted least square mean and variance adjusted (WLSMV) was used to estimate the model’s parameters, whilst the robust standard errors were used. The absolute fit (i.e., root mean square error of approximation [RMSEA], standardized root mean square residual [SRMR], goodness of fit index [GFI]), and the incremental fit (i.e., adjusted goodness of fit index [AGFI], comparative fit index [CFI], Tucker-Lewis index [TLI], normed fit index [NFI]) were observed for the model fit estimation. An excellent model fit is determined when at least two of the four fit indices exceed the following thresholds: RMSEA ≤ 0.05; SRMR ≤ 0.05; CFI ≥ 0.95; NNFI ≥ 0.95 [30]. Hu and Bentler [31] proposed also the thresholds for the following indices: TLI ≥ 0.95, CFI ≥ 0.90, AGFI ≥ 0.90 and GFI ≥ 0.95. We also calculated the percentage of subjects achieving the highest (floor effect) and the lowest (ceiling effect) scores on each question of HADS. Finally, chi-square tests were conducted to determine differences by gender and age (grouped as <45 years, 45–59 years, 60–69 years, and ≥70 years).

## 3. Results

### 3.1. Participants

From 2000 patients randomly selected and invited to participate from the involved hospitals, 1969 (mean age: 61, SD: 16 years, 46.5% women) were finally included. Each patient reported a mean of 2.2 (SD 0.8) COVID-19 onset symptoms at hospital admission. The most prevalent COVID-19 associated onset symptoms at hospital admission were fever (74.6%), dyspnoea (31.5%) and myalgia (30.7%). All participants were assessed a mean of 8.4 months (SD, 1.5) after hospital discharge. The features of the total sample are shown in Table 1 [16,17,18,19,20,21].

### 3.2. General Data

The mean time for fulfilling HADS was 65 (SD 12) seconds. All questions were answered by participants. The mean HADS-A score was 4.9 (SD 5.2), whereas the HADS-D score was 4.7 (SD 4.8). The percentage of data at ceiling (nil dysfunction) ranged from 1.99% to 13.74% and the percentage of data at floor (maximal dysfunction) ranged from 43.05% to 77.77% (Table 2).

**Figure 1:**
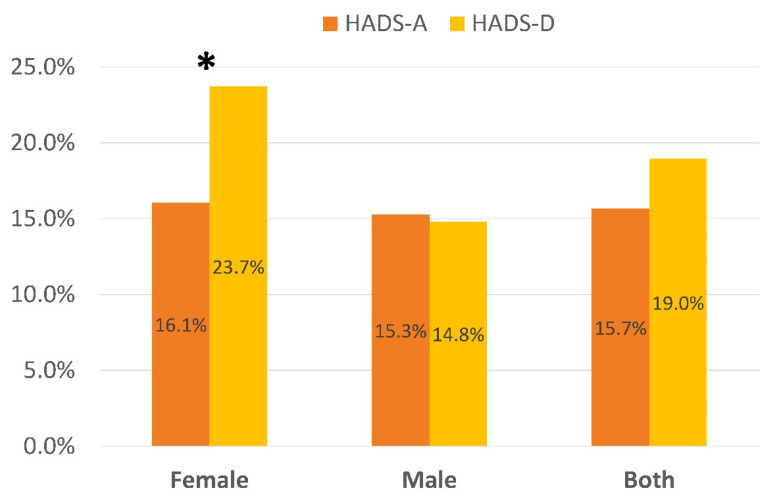
Distribution of the percentage of women and men exhibiting anxiety (HADS-A ≥ 8 points) and depressive (HADS-A ≥ 8 points) symptoms according to the Hospital Anxiety and Depression Scale (HADS) * Significant differences between female and male (*p* < 0.01).

**Figure 2:**
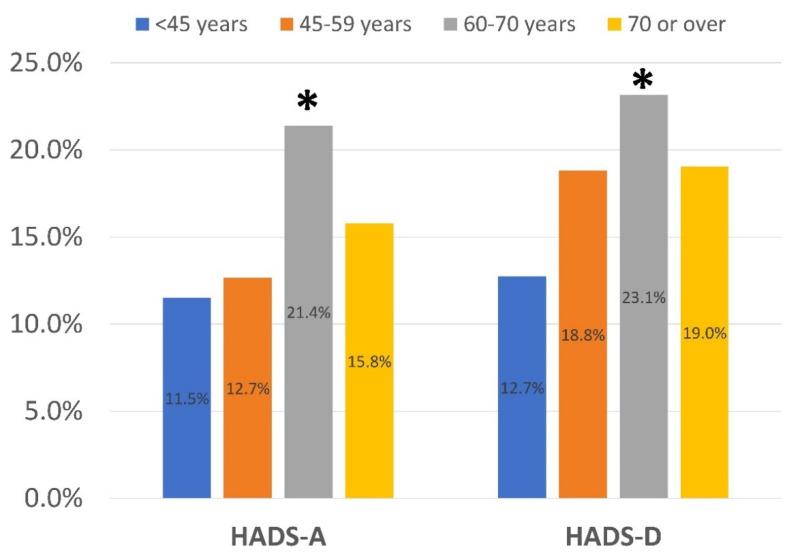
Distribution of the percentage of individuals exhibiting anxiety (HADS-A ≥ 8 points) and depressive (HADS-D ≥ 8 points) symptoms according to the Hospital Anxiety and Depression Scale (HADS) by age group. * Significant differences by age group (*p* < 0.01).

A greater (*p* < 0.001) proportion of women exhibited depressive levels (HADS-D ≥ 8 points) as compared to men. No differences (*p* = 0.637) in the proportion of women and men reporting anxiety levels (HADS-A ≥ 8 points) existed (Figure 1). Significant differences across the age-groups were observed for the proportion of those with anxiety (*p* < 0.001) and depressive (*p* = 0.003) levels: individuals aged 60–70 years reported higher anxiety and depressive levels than the other age-groups (Figure 2).

### 3.3. Reliability and Internal Consistency

The item-internal consistency ranged from 0.520 to 0.888 for HADS-A items and from 0.659 to 0.833 for HADS-D items, suggesting moderate to excellent consistency (Table 2). The correlation of each of the items with its hypothesized scale was smaller than its correlation with the other scale; accordingly item-discriminant validity was not supported by current data. In fact, item-reliability coefficients were smaller than the correlation coefficient between the HADS-A and HADS-D scales (r: 0.878)

The Cronbach’s α value of the HADS-A score was 0.890 whereas the Cronbach’s α of the HADS-D score was 0.856, supporting good internal consistency (Table 2). Additionally, the Omega coefficient of the HADS-A score was 0.911 whereas the Omega coefficient of the HADS-D score was 0.821 (Table 2).

### 3.4. Construct Validity

**Figure 3:**
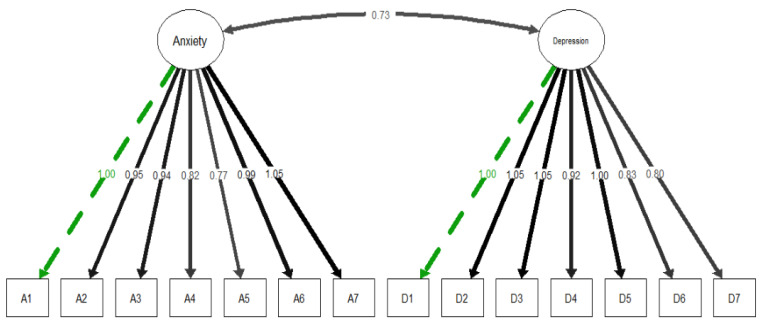
Structural equation modeling (SEM) of the Hospital Anxiety and Depression Scale (HADS) in COVID-19 survivors. Dotted line indicates fixed loading. Items of anxiety subscale (A1, A2, A3, A4, A5, A6, A7). Items of depression subscale (D1, D2, D3, D4, D5, D6, D7).

Table 3 details the tested measurement model and associated regression weights of the CFI. Five out of the seven fitness indexes were excellent: CFI = 0.969, NNFI = 0.963; TLI = 0.963; AGFI = 0.951; GFI = 0.972), suggesting that that fit for the measurement model was excellent. However, two fitness indexes did not satisfy their excellent thresholds: RMSEA = 0.217, SRMR = 0.182. Figure 3 presents structural equation modeling (SEM) of HADS in previously hospitalized COVID-19 survivors, and demonstrates that the two subscales (anxiety and depression) were positively and significantly correlated with each other. The factor loading of each item of HADS was also acceptable.

The specific questions to which these items refer are as follows. A1: I feel tense or ‘wound up’; A2: I get a sort of frightened feeling as if something awful is about to happen; A3: Worrying thoughts go through my mind; A4: I can sit at ease and feel relaxed; A5: I get a sort of frightened feeling like ‘butterflies’ in the stomach; A6: I feel restless as I have to be on the move; A7: I get sudden feelings of panic; D1: I still enjoy the things I used to enjoy; D2: I can laugh and see the funny side of things; D3: I feel cheerful; D4: I feel as if I am slowed down; D5: I have lost interest in my appearance; D6: I look forward with enjoyment to things; D7: I can enjoy a good book or radio or TV program.

## 4. Discussion

Several symptoms perceived after the acute phase of SARS-CoV-2 infection, i.e., post-COVID, are physical; however, psychological or emotional disturbances are also present [3,4]. The heterogeneity manifestations of long COVID supports the need for an assessment covering organic and emotional aspects. HADS is a PROM commonly used for evaluating anxiety and depressive symptoms in different population including COVID-19 survivors [3,4]. The current study evaluated the psychometric properties of HADS in previously hospitalized COVID-19 survivors with long COVID. The results reveal that HADS has appropriate floor and ceiling effects, was internally consistent and reliable, and has overall construct validity for assessing anxiety and depressive symptoms in people with long COVID. These psychometric properties are similar to data published on the general population [12] and in healthcare workers who were in the frontline during the COVID-19 outbreak [14]. Accordingly, HADS could be considered as a comprehensive and an easy PROM to be applied in patients with long COVID since it takes around 60 sec to be fulfilled.

HADS was originally created as two subscales focusing on anxiety and depressive symptoms [11]. Although the current study did not attempt to identify the presence of anxiety and depressive symptoms in our sample of individuals with long COVID, current evidence supported that a cut-off value ≥ 7 points on HADS-D had a sensitivity of 0.82 and specificity of 0.78 whereas a cut-off value ≥ 8 points showed a sensitivity of 0.74 and a specificity of 0.84 for identifying depressive symptoms [32]. We identified that almost 20% of our sample experienced symptoms of depression based on the cut-off value of HADS-D ≥ 8 points. In fact, our study found both scales exhibited overall good psychometric data in people with long COVID. However, we should recognize that, although most goodness of fit indexes were excellent, the RMSEA was high, suggesting that the model contained some sort of misspecification, e.g., redundancy among items. These data are similar to those previously reported by Norton et al. [33]. These authors pooled data from 28 different samples obtained from published studies concerning the latent structure of HADS and conducted several analyses [33]. Current evidence supports that a bifactor structure provides the most acceptable empirical explanation for the HADS correlation structure; however, due to the presence of a strong general factor, HADS does not provide a good separation between symptoms of anxiety and depression, but it is likely that this is a problem for most instruments where symptoms of anxiety and depression are jointly measured [33]. In line with this situation, LoMartire et al. have recently reported that rather than using both subscales of anxiety and depression separately, HADS as a single score is also a valid and reliable PROM of overall emotional distress, at least in people with chronic pain [34].

Women exhibited more depression, but not anxiety, symptoms than men, supporting current assumptions that females exhibit more emotional post-COVID symptoms [19,35]. The lack of gender differences in anxiety in the current study may be related to the fact that our sample included individuals hospitalized during the first wave of the COVID-19 outbreak. Recent studies have reported that depression and anxiety symptoms were more pronounced during the first wave of the COVID-19 outbreak than during the second [36] or later [37] waves. We also observed that individuals aged 60–70 years reported more anxiety and depression than other age-groups. Since patients from our study were hospitalized during the first wave of the COVID-19 outbreak, it is possible that individuals in this age group perceived the first outbreak as more of a threat than other age-groups due to a higher self-perception of frailty against SARS-CoV-2 virus. Nevertheless, this hypothesis should have been more evident in the oldest group. Future studies should test this hypothesis.

The results from this study should be considered according to their strengths and limitations. The main strength consists of the inclusion of a large sample of COVID-19 survivors from different centers and with a long-term follow-up after hospital discharge. However, the main limitation is the inclusion of only hospitalized COVID-19 survivors. We do not know if HADS would exhibit similar psychometric data in non-hospitalized subjects, where emotional aspects could be less relevant than in those hospitalized. Additionally, although we assessed patients at a long-term follow-up period after hospitalization, we did not collect the emotional status of these patients before acute infection or during the first months after hospitalization. Studies investigating the longitudinal evolution of anxiety or depressive symptoms in this population are needed. In fact, longitudinal evolution of depressive and anxiety symptoms during the COVID-19 era is conflicting. Gambin et al. found that the increased anxiety/depressive levels occurring at the early stages of the first COVID-19 lockdown decreased in a small proportion of subjects, being resilient or chronic in most of them [38]. On the contrary, Saunders et al. observed that most individuals with anxiety or depression symptoms evolved positively during the first week after the COVID-19 lockdown [39]. The only study investigating the longitudinal evolution of anxiety and depressive symptoms in subjects with long COVID found that post-COVID anxiety and depressive symptoms tended to slowly recover during the following five years after the SARS-CoV-2 infection [40]. Finally, it is important to consider limitations related to the methodological design of the study. This study only analyzed the psychometric properties of HADS in a specific population such as individuals with long COVID. Although we presented properties including internal consistency, item-internal consistency, item-discriminant validity and construct validity, we did not compare HADS with other PROMs and we did not provide test-retest reliability.

## 5. Conclusions

This study suggests that HADS exhibits overall good psychometric properties, appropriate floor and ceiling effects, good internal consistency and reliability, and construct validity, to be used as a PROM to evaluate the presence of anxiety and depressive symptoms in previously hospitalized COVID-19 survivors; nevertheless, values of some indexes suggest that some items can be redundant. Additionally, other PROMs evaluating physical impairments should be used complementarily with HADS for evaluating long COVID patients. The systematic use of HADS in future studies of long COVID could help worldwide comparisons of the presence of mood disorders in this population.

## Figures and Tables

**Table 1:** Clinical/Hospitalization Data and Post-COVID Symptoms (*n* = 1969).

**Age, mean (SD), years**	61 (16)
**Gender, male/female (%)**	1054 (53.5%)/915 (46.5%)
**Weight, mean (SD), kg.**	75 (15)
**Height, mean (SD), cm.**	165 (16.5)
**Medical co-morbidities**	
Hypertension	514 (26.1%)
Diabetes	236 (12.0%)
Cardiovascular Disease	234 (11.9%)
Asthma	126 (6.4%)
Obesity	88 (4.5%)
Chronic Obstructive Pulmonary Disease	77 (3.9%)
Stroke	38 (2.0%)
Rheumatological Disease	31 (1.6%)
Other (Cancer, Kidney Disease)	332 (16.9%)
**Main Symptoms at hospital admission, *n* (%)**	
Fever	1469 (74.6%)
Dyspnea	620 (31.5%)
Myalgia	604 (30.7%)
Cough	549 (27.9%)
Headache	332 (16.9%)
Diarrhea	210 (10.7%)
Anosmia	167 (8.5%)
Ageusia	145 (7.35%)
Throat Pain	102 (5.2%)
Vomiting	55 (2.8%)
**Stay at the hospital, mean (SD), days**	11.3 (11.4)
**Intensive Care Unit (ICU) admission**	
Yes/No, *n* (%)	130 (6.6%)/1839 (93.4%)
**Persistent post-COVID symptoms, *n* (%)**	
Fatigue	1206 (61.3%)
Dyspnea at exertion	1054 (53.5%)
Pain Symptoms	887 (45.1%)
Loss hair	470 (23.9%)
Dyspnea at rest	459 (23.3%)
Memory loss	341 (17.3%)
Skin Rashes	236 (12.0%)
Brain fog	189 (9.6%)
Concentration loss	140 (7.1%)
Tachycardia-Palpitations	140 (7.1%)
Gastrointestinal Disorders	133 (6.75%)
Ocular/Vision Disorders	116 (5.9%)
Anosmia	80 (4.05%)
Ageusia	53 (2.7%)
Throat Pain	50 (2.5%)
Diarrhea	49 (2.5%)
Voice problems	35 (1.8%)

**Table 2:** Internal Consistency, Discriminant Validity, Floor and Ceiling Effect of each Item of the Hospital Anxiety and Depression Scale (HADS) in COVID-19 survivors experiencing long COVID.

	Item-Internal Consistency	Item-Discriminant Validity	Cronbach’s Alpha	Omega	Floor Effect	Ceiling Effect
I feel tense or ‘wound up’	0.888 ***	0.832 ***	0.890 (HADS-A)	0.911 (HADS-A)	43.05%	12.46%
I get a sort of frightened feeling as if something awful is about to happen	0.845 ***	0.768 ***			52.97%	11.62%
Worrying thoughts go through my mind	0.857 ***	0.788 ***			43.40%	11.45%
I can sit at ease and feel relaxed	0.574 ***	0.461 ***			70.52%	3.38%
I get a sort of frightened feeling like ‘butterflies’ in the stomach	0.520 ***	0.419 ***			77.77%	1.99%
I feel restless as I have to be on the move	0.873 ***	0.812 ***			55.57%	11.29%
I get sudden feelings of panic	0.795 ***	0.700 ***			73.74%	13.74%
I still enjoy the things I used to enjoy	0.744 ***	0.649 ***	0.856 (HADS-D)	0.821 (HADS-D)	57.47%	3.75%
I can laugh and see the funny side of things	0.743 ***	0.652 ***			63.86%	2.26%
I feel cheerful	0.833 ***	0.744 ***			51.26%	12.24%
I feel as if I am slowed down	0.701 ***	0.565 ***			55.04%	11.11%
I have lost interest in my appearance	0.737 ***	0.604 ***			63.36%	13.00%
I look forward with enjoyment to things	0.738 ***	0.621 ***			54.50%	8.03%
I can enjoy a good book or radio or TV program	0.659 ***	0.559 ***			66.38%	2.01%

*** *p* < 0.001.

**Table 3:** Confirmatory factor analysis of each item of the Hospital Anxiety and Depression Scale (HADS) in COVID-19 survivors experiencing Long COVID. Factor loadings.

LHS	RHS	Coef	SE	Pval	Type
**Anxiety**	**A1**	**1.000**			**Latent**
Anxiety	A2	0.953	0.009	<0.001	Latent
Anxiety	A3	0.945	0.009	<0.001	Latent
Anxiety	A4	0.822	0.014	<0.001	Latent
Anxiety	A5	0.769	0.018	<0.001	Latent
Anxiety	A6	0.987	0.008	<0.001	Latent
Anxiety	A7	1.047	0.009	<0.001	Latent
**Depression**	**D1**	**1.000**			**Latent**
Depression	D2	1.047	0.008	<0.001	Latent
Depression	D3	1.049	0.009	<0.001	Latent
Depression	D4	0.919	0.013	<0.001	Latent
Depression	D5	1.004	0.011	<0.001	Latent
Depression	D6	0.829	0.012	<0.001	Latent
Depression	D7	0.799	0.014	<0.001	Latent
A1	A1	0.158			vCov
A2	A2	0.236			vCov
A3	A3	0.249			vCov
A4	A4	0.431			vCov
A5	A5	0.503			vCov
A6	A6	0.180			vCov
A7	A7	0.078			vCov
D1	D1	0.200			vCov
D2	D2	0.124			vCov
D3	D3	0.120			vCov
D4	D4	0.324			vCov
D5	D5	0.195			vCov
D6	D6	0.450			vCov
D7	D7	0.490			vCov
Anxiety	Anxiety	0.842	0.010	<0.001	vCov
Depression	Depression	0.800	0.010	<0.001	vCov
Anxiety	Depression	0.729	0.010	<0.001	vCov

Abbreviation: LHS—left hand side of equation; RHS—right hand side of equation; Coef—unstandardized coefficient; SE—standard error; Pval—*p* values; vCov—variance covariance. Bold indicates fixed loading factor.

## Data Availability

Not applicable.
